# The *Children – Sit Less, Move More* (C-SLAMM) pilot intervention: Feasibility and acceptability of a multi-component school and home-based intervention to promote physical activity

**DOI:** 10.1371/journal.pone.0335933

**Published:** 2025-11-19

**Authors:** Sarah Nally, Angela Carlin, Alison M. Gallagher, Jason J. Wilson, Ian M. Lahart, Jo Salmon, Marie H. Murphy

**Affiliations:** 1 Centre for Exercise Medicine, Physical Activity and Health, Sports and Exercise Sciences Research Institute, Ulster University, Belfast, United Kingdom; 2 Trinity Centre for Practice and Healthcare Innovation, School of Nursing and Midwifery, Trinity College Dublin, Dublin, Ireland; 3 Nutrition Innovation Centre for Food and Health (NICHE), Biomedical Sciences Research Institute, Ulster University, Coleraine, United Kingdom; 4 Faculty of Education, Health and Wellbeing, University of Wolverhampton, Walsall, United Kingdom; 5 Institute for Physical Activity and Nutrition (IPAN), School of Exercise and Nutrition Sciences, Deakin University, Burwood, Victoria, Australia; 6 Physical Activity for Health Research Centre (PHARC), Institute for Sport, Physical Education and Health Sciences, University of Edinburgh, Edinburgh, United Kingdom; University of Stirling, UNITED KINGDOM OF GREAT BRITAIN AND NORTHERN IRELAND

## Abstract

**Background:**

A high proportion of primary school children in Northern Ireland (NI) are insufficiently active. In response, an intervention adapted from the *TransformUs* programme was established to promote physical activity (PA) and reduce sedentary behaviour (SB). This study aimed to assess the feasibility of recruitment and retention, data collection procedures, intervention acceptability and explore preliminary effectiveness on children’s PA and SB levels.

**Methods:**

The *Children – Sit Less, Move More (C-SLAMM*) intervention integrated behavioural, pedagogical, and environmental strategies across classroom, school, and home settings. Eight primary schools were recruited and randomly assigned (1:1) to either the intervention or control. Feasibility measures included school and participant recruitment, retention and completion rates. Acceptability was assessed using weekly diary logbooks, fidelity checklists and qualitative methods (write and draw activity, focus groups, interviews). Children (aged 7–9 years) wore activPAL accelerometers continuously for 7 days at baseline and post-intervention (Week 8) to measure time spent sitting, standing, and stepping.

**Results:**

A total of 194 consent forms were distributed. Of the 162 children who consented (84% response rate), 76 (46.9%) met the valid wear-time criteria at both baseline and follow-up. Intervention delivery varied across schools, impacting fidelity. Qualitative analysis revealed four themes: (1) engagement, (2) positive aspects of *C-SLAMM* intervention, (3) barriers to intervention delivery, and (4) recommendations for improvement. Children and teachers generally found the intervention acceptable, though barriers included limited parental support, inadequate classroom space and time constraints. There were no significant differences in sitting time (β = −6.5 minutes/day; 95%CI: −36.4, 23.4), standing or stepping time between groups. Nevertheless, the intervention was seen as enhancing classroom experiences for both children and teachers.

**Conclusions:**

The *C-SLAMM* intervention was well-received and shows promise as an acceptable approach to reduce sitting time and promote PA. Further refinement of data collection methods is needed before progressing to a pilot trial.

**Trial registration:**

ClinicalTrials.gov, NCT05854355 (submitted on the 30^th^ of March 2023).

## Introduction

Children spend most of their waking hours engaged in sedentary behaviour (SB) [1,2]. High levels of SB has been linked to adverse cardiometabolic heath risk markers, reduced self-esteem, and poor academic performance in children [3,4]. Regular physical activity (PA) is associated with many health benefits for children, including improved cardiometabolic health, the promotion of healthy weight and increased psychological well-being [5,6]. Recent evidence suggests that the effects of SB and PA are not as *‘independent’* as previously considered and may interact to influence children’s health outcomes [7,8]. Both movement behaviours (e.g., PA and SB) are associated with various health outcomes in children [9].

Despite these known benefits, the latest global PA figures indicate that children’s PA levels remain a serious public health concern [10]. Global data from the *Global Matrix 4.0* reveals a concerning trend, with only 27–33% of children (aged 5–17 years) worldwide meeting the recommended 60 minutes of moderate-to-vigorous physical activity (MVPA) per day [10]. This indicates a decline from the *Global Matrix 3.0*, where 34–46% of children met the PA guidelines [11]. As a result, the public health recommendation to “*sit less, move more*” is becoming more widespread, highlighting the urgent need to develop interventions that can increase daily PA and reduce SB. In Northern Ireland (NI), national guidelines recommend that children and adolescents (6–17 years) engage in at least 60 MVPA per day [12]. Additionally, children are advised to limit SB, particularly the amount of recreational screen time per day [13]. Yet, despite the national mandate and policy support, nationally representative data indicates that 79% of primary schoolchildren (aged 9–11 years) in NI fail to meet PA recommendations and spend prolonged periods sitting [14,15].

Primary schools offer an invaluable opportunity to accumulate PA [16] and reduce SB [17], as they can target all pupils, irrespective of their socioeconomic status, providing access to those who may benefit most [18,19]. Schools offer numerous opportunities for increasing PA, such as during recess/lunchtime [20], physical education (PE) lessons [21], classroom-based interventions [18,22]; and after-school programmes [23,24]. Despite these opportunities, school-based PA interventions have shown mixed results in improving PA outcomes in primary-school children [25]. Current evidence suggests that multicomponent interventions using a whole-school approach, aimed at reducing SB and promoting PA throughout the entire school day, have the greatest potential for positive impact on time spent in MVPA and SB [17,26,27]. Despite a growing evidence base, there is limited understanding of how to replicate these interventions across diverse educational settings [28]. Furthermore, a school-based PA intervention that is developed and proven effective in one educational or national context may not yield the same results elsewhere, highlighting the need for further research into the generalisability and adaptability of school-based PA interventions [29].

One example of a recent intervention that has been successful in increasing PA and reducing SB in Australian primary school pupils is the *TransformUs* intervention [30,31]. *TransformUs* was developed to determine the impact of strategies to promote children’s PA and/or reduce SB, on health and behavioural outcomes in Australian children (aged 8–9 years) [30]. The *TransformUs* cluster-randomised controlled trial (cRCT) included three intervention arms: (1) targeted reductions in SB (SB-I group), (2) increases in PA (PA-I group), and (3) a combination of both (SB + PAI group) across the school day which were incorporated through a mixture of educational, pedagogical, behavioural, and environmental strategies to integrate movement into class lessons, recess/lunchtime, and homework [30]. *TransformUs* was deemed appropriate for the school-setting [32], effectively reduced children’s SB [31] and demonstrated that the *“break up your sitting*” statement may be more impactful than the “*move more”* statement in schoolchildren aged 8–9 years [33]. While *TransformUs* has been effective in Australia, no similar low-cost multi-component PA and SB intervention has been implemented in NI.

There is a need for researchers to consider a context-specific intervention approach to school-based PA intervention design [34]. Given the high levels of physical inactivity among primary-school children in NI and the lack of similar multi-component intervention in the region, researchers adapted the existing *TransformUs* intervention for potential delivery across primary schools in NI. Local contextual differences, including variations in the education system, social dynamics, and environmental factors influencing PA, render wholescale replication in NI inappropriate [34,35]. Therefore, key strategies from the *TransformUs* intervention were adapted to develop a context-specific school- and home-based intervention, called the *Children – Sit Less, Move More* (C-SLAMM) intervention.

In line with the MRC framework for developing and evaluating complex interventions [36], the initial phase of complex intervention research involves the development or adaption of an existing intervention [36]. A formative qualitative exploration was conducted to gain a detailed understanding of NI school children’s (aged 7–9 years) perceptions of context-specific PA, alongside the barriers and enablers influencing their ability to lead a physically active lifestyle [37]. Through in-depth semi-structured interviews and focus groups, valuable contextual information was gathered, highlighting the perspectives of children, their parents’ and teachers’ regarding school- and home-based PA, as well as the meanings children ascribe to PA [37,38]. Subsequently, this formative work coupled with resources from the *TransformUs* intervention, informed the development of the context-specific *C-SLAMM* intervention.

The aim of this pilot study was to assess the feasibility and acceptability of implementing this multicomponent intervention (*C-SLAMM*), adapted from the efficacious *TransformUs* programme to reduce sitting time and increase PA in Primary 4 (P4) and Primary 5 (P5) school children (aged 7–9 years) in NI. The rationale behind conducting a pilot study, and not an effectiveness study, was to focus on potential intervention development, refinement and to address uncertainties around the feasibility of intervention methods [39,40]. Specifically, the study aimed to assess the feasibility of recruiting and retaining children, evaluate the appropriateness of data collection procedures, determine the acceptability of intervention implementation and explore preliminary effectiveness on children’s PA and SB levels.

## Methods

### Study design

A mixed-method two-armed pilot study was conducted between 1^st^ of September 2021 and 12^th^ of May 2022. Due to COVID-19 pandemic restrictions, the intervention used a phased approach to recruitment (Phase 1 and Phase 2) (see S1 File). To control for potential contamination between intervention and control schools, randomisation was carried out on a cluster level [41]. Schools were the unit of allocation (cluster), and individuals (children aged 7–9 years) were the unit of analysis. Randomisation was conducted using a computer-based random number generator after baseline data were collected (October 2021) and performed by a researcher not involved in data collection. Given the nature of the intervention, blinding of schools, participants, and researchers involved in data collection were not possible following randomisation. This trial has been registered prospectively with ClinicalTrials.gov under the number NCT05854355.

The intervention was conducted in line with MRC guidelines developing and evaluating complex interventions [36]. Where relevant, the Consolidation Standards of Reporting Trials (CONSORT) checklist for pilot trials informed the study design [42]. Ethical approval for this study was obtained from Ulster University Ethics Committee (REC/21/0027).

### Sample size estimation for feasibility outcomes

In line with recommendations for feasibility and pilot studies [40,42], we estimated a sample size that reflected the target population and setting. The recruitment target was set at eight primary schools, with an estimated minimum of 15 primary-school children per school (approximately half the size of a typical class in NI), resulting in a total of at least 120 children. This target was consistent with sample sizes used in similar pilot studies [43,44].

### Eligibility and recruitment

In September 2021, a convenience sample of primary schools in NI were invited to take part in the study [45]. Eleven primary schools across three geographical areas (counties) in NI, specifically: Antrim, Armagh, and Derry/Londonderry, were invited to take part in the study via e-mail/telephone. Eight primary schools agreed to participate in the study. Following school principal consent, information packs and consent forms were distributed to all eligible teachers and parents of children in P4 (7–8 years old) and/or P5 (8–9 years old). Written informed consent was obtained for all child (parental consent and child assent) and teacher participants. Children were required to provided written assent prior to the baseline measurement and were excluded from the study if they had any medical condition that limited their participation in a PA intervention. Control schools were informed via email of their selection and agreed to continue with their usual timetabled number of breaks and physical education (PE) lessons without any additional time allocated for PA participation. Data relating to the recruitment (number of schools and participants approached, excluded and consented) and retention (number of participants who withdrew, were lost to follow-up or who provided data) were recorded.

### Intervention

The *C-SLAMM* intervention was an 8-week school- and home- intervention aimed at reducing sitting time and increasing PA in primary-school children. The intervention incorporated behavioural, pedagogical, and environmental strategies within the classroom, school, and home setting. The low-cost intervention was designed to be delivered by generalist teachers (i.e., teachers who did not have specific expertise in teaching PE). The intervention consisted of six key components (outlined in Table 1): (i) health lessons incorporating key PA/SB messages, (ii) active lessons, (iii) breaks from sitting, (iv) promotional signage and class sets of sports/PA equipment for use during class time, lunchtime and breaktime, (v) active homework, and (vi) parent newsletters promoting PA/reducing sitting time. The *C-SLAMM* intervention content was adapted from the *TransformUs* programme [30], which was guided by the social cognitive theory [46], behavioural choice theory [47] and ecological systems theory [48]. The research team had full access to all the *TransformUs* materials [30] and tailored them to the NI context. Adaptations included revising language and content to align with local terminology, regional references, seasonal variations, and replacing Australian-specific animals such as ‘*koala’* with contextually relevant examples.

**Table 1:** Components of the *Children – Sit Less Move More* (C-SLAMM) intervention.

Intervention component	Target domain	Duration	Frequency	Description
**Breaks from sitting**	Classroom	2 minutes	x6/day	Every 2-hour teaching block were to be interrupted every 30 minutes with a 2-minute guided light-intensity activity break. A novelty timer was given to each class so that teachers could monitor 2-minute standing breaks and every 30 minutes of sitting class time.
**Active lesson**	Classroom	30 minutes	x1 class lesson/day	Teachers were asked to modify the delivery of at least one class lesson so that children completed the lesson standing up. Any lesson were provided with a suite of standing lesson delivery methods that could be modified to any class topic.
**Active homework**	Family	Varied	x1/week	Homework tasks were modified to incorporate PA, and children were encouraged to complete these tasks with their parents (e.g., go for a walk with their parents and write about where they went and what they saw; mathematics homework using their stride as the unit of measurement).
**Health lesson**	Classroom	30-40 minutes	Every 2 weeks	Four complete lesson plans were provided. Health lessons provided four key learning messages: • Physical activity, sitting time and health • Self-management of sitting time and PA for children • Decision making (Choosing to move or sit) • Active families
**Newsletters for home**	Family	N/A	Every 2 weeks	Newsletters were sent home bi-weekly to parents providing tips on promoting their child’s PA and how to decrease SB. Newsletters were linked to the key learning messages from the health lesson component.
**Active environment**	Classroom and/or school	N/A	N/A	Schools were provided with a low-cost box of PA and sports equipment (£200 per box) to facilitate play and PA for children to use during lunch breaks, and teachers could provide encouragement and support for active games. Six electric height adjustable sit-stand desks per classroom. Provision of sporting equipment and signage (indoor only) to promote PA. Each sit-stand desk accommodated two children.

N/A: not applicable, PA: physical activity, SB: sedentary behaviour.

During the intervention, PA was promoted and encouraged throughout breaktime and lunch breaks. Each class was provided with six electric height adjustable sit-stand desks (*Alpha, DF32 Model, UK)* allowing children to rotate between sitting and standing during learning activities at designated *‘standing stations’*. The sit-stand desks were installed in the intervention classroom following baseline measurements and were designed to accommodate up to two children per desk. Traditional classroom chairs were retained for use with the new desks. Prior to the start of the intervention, children and teaching staff were shown how to adjust desk height by the research team.

Teachers were asked to use a weekly logbook to track implementation of the intervention components. The intervention groups received support from the researcher (SN) with biweekly visits during the intervention period. Within the control group, teachers continued with normal lesson delivery with no change to the classroom environment. To support implementation, teachers completed a 45-minute face-to-face training delivered by SN, and were provided with *C-SLAMM* intervention materials, lesson planning guides and access to an online teacher training handbook. Teacher training covered what resources were available, how to utilise the resources and how often each intervention component was to occur across the school week. Schools randomised to control were offered information on the intervention and associated materials at the end of the intervention.

### Measures

The feasibility of the intervention was assessed using measures of eligibility and recruitment, intervention implementation (e.g., resources and delivery), and the acceptability of data collection procedures. Potential efficacy was assessed using both quantitative and qualitative methods, as summarised in S2 File.

### Qualitative measures

To assess the acceptability of the intervention, write and draw activity and focus groups were conducted with children, while semi-structured interviews were undertaken with teachers during Week 9.

### Write and draw activity with children

In each intervention school (*n* = 4), the researcher (SN) facilitated two groups of six children each in write and draw and focus group activities (*n* = 48 in total). Participants were randomly selected using a number generator, and verbal assent was obtained from all children before starting the activities. The write and draw activity was conducted prior to the focus groups and comprised of a one-sided sheet of paper divided into two interrelated sections: one for written descriptions and the other for drawings (see S3 File for a copy of the write and draw activity).

To begin, the researcher (SN) presented visual illustrations of the *C-SLAMM* intervention components. Visual illustrations included: activity cards (used in a health lesson), a newsletter, a timer, a sit-stand desk, classroom box of PA equipment and active homework. These illustrations served as reminders of each intervention component. Following this, children independently completed the write and draw activity, expressing their perceptions and experiences of the *C-SLAMM* intervention. The first section asked children to describe their favourite component of the intervention, while the second asked them to draw a picture of themselves engaging in that activity.

### Focus groups with children

Semi-structured focus groups were conducted after the write and draw activity, with six children per group (*n* = 48, 8 focus groups in total). A semi-structured guide was developed and used to ensure consistency across the groups (a copy of the focus group guide is provided in S2 File). The questions were devised to elicit children’s perceptions of the *C-SLAMM* intervention components. All data were anonymised, and any comments or observations relating to specific individuals or schools were removed to maintain confidentiality.

### Semi-structured interviews with teachers

Face-to-face interviews (one per teacher) were completed to explore teachers’ perceptions of the appropriateness and barriers/facilitators of widespread integration of the *C-SLAMM* intervention (a copy of interview guide is given in S3 File). Interviews took place in a quiet, private area of the school at a convenient time for the teachers.

### Teacher logbook and fidelity checks

To explore feasibility of the intervention, all participating teachers were asked to complete a weekly logbook to report on the delivery of the intervention components. One logbook was provided to each intervention school and teachers were asked to record entries as and when possible. The logbooks collected information on adherence, exposure and, to some extent, intervention differentiation (e.g., variation in active homework activities across different schools). Additionally, a researcher (SN) conducted weekly fidelity checks via in-person visits or phone calls, based on the teacher’s preference and availability. During these interactions, the researcher completed the fidelity checklist and discussed: (1) which components of the intervention had been implemented during the school week, (2) any challenges the teacher encountered, and (3) whether the logbook had been completed.

### Child-level outcomes

#### Sitting and physical activity.

Time spent in different postures (sitting, standing and stepping) during school hours and across the full week were assessed using the activPAL3 monitor *(PAL Technologies Ltd, Glasgow, UK*). Participants were asked to wear the monitor continuously on the anterior aspect of the right thigh for 24 hours/day, for seven consecutive days at baseline (Week 0) and during the final week of the intervention (Week 8). Devices were attached at school using a waterproof dressing [using a nitrile sleeve and hypoallergenic Hypafix® (BSN medical, Hull, UK). Participants received verbal and written instructions, along with a pre-recorded video demonstrating its use [49]. Participants were advised to remove the device for swimming, bathing, contact sports or if any skin irritation occurred [49]. Participants were also provided a diary and instruction sheet to document time in bed and any periods of non-wear with the help of their parent/guardian. As an incentive, participants who completed baseline and follow-up measurements were given rewards of low monetary value (e.g., a ball, stickers and pencils).

All activPAL data were downloaded (PAL files using manufacturer proprietary software (activPAL Professional v.7.2.29) in 15-s epochs and processed using a customised Microsoft Excel macro. The PAL files were visually inspected once downloaded within the activity summary feature of the software as a basic compliance check. Periods of non-wear and sleep time were excluded from the analyses using manufacturer proprietary software, supplemented with cross-checking against participants’ diary entries. The total non-wear time for each day was summed. Total sitting, standing and stepping time; total number of steps and number of sit to stand transitions during the 7-day period were determined. Wear time compliance was set at ≥10h/day for at least 2 days [50,51]. To assess the impact of the intervention on school-time activity levels, a school-time filter (09:00–15:00) was applied during data analysis, designed to capture the time children spent sitting, standing and stepping during school hours [52]. For this school-time analysis, the same daily wear-time compliance (≥10h/day for at least 2 days) was required [50,51], with an additional criterion specifying valid school wear time as having at least 4.5 hours (270 minutes) of data during school time [53,54].

#### Anthropometrics.

Height and weight were measured (to the nearest cm/kg) at baseline and follow-up using a portable stadiometer (Seca UK, Birmingham, UK) and portable electronic weighing scales (Seca model 887). Height and weight were measured barefoot. Body Mass Index (BMI) was calculated (as kg/m^2^) from height and weight measurements.

#### Health-Related Quality of Life.

Children’s health-related quality of life (HRQoL) was assessed at baseline (Week 0) and during the final week of the intervention (Week 8) using the validated self-report Kidscreen-27 questionnaire [55] which consists of 27 items across five dimensions: physical wellbeing, psychological wellbeing, parents/guardians’ relations and autonomy, social support and peers, and the school environment. Responses to each question were recorded using 5-point Likert scales (1 = never to 5 = always) of certain behaviours, or the intensity of an attitude (1 = not at all to 5 = extremely). The Kidscreen-27 was analysed using the methodology described in the Kidscreen administration manual [56].

### Data analysis

For qualitative analyses, focus groups and semi-structured interviews were audio-recorded, transcribed verbatim and anonymised. To reduce possible researcher bias triangulating of the three data sources was undertaken; namely, focus group transcripts, interview transcripts and child drawings [57,58]. Verbatim quotes and drawings from the children’s participatory focus groups and teachers’ semi-structured interviews were extracted to exemplify representation of the participants’ experiences and perceptions.

Qualitative data were managed using NVivo12 (Version 12.6.0; QSR International Pty Ltd., Victoria, Australia). Thematic analysis, using Braun and Clarke’s phases [59], guided the coding process. Transcripts were systematically coded and key themes were developed. Content analysis was used to analyse the write and draw activity. A second researcher served as a “*critical friend*” independently reviewing a subsample of transcripts, offering alternative interpretations and prompting reflection to ensure rigor. Researchers critically discussed and reached consensus on all steps and outcomes in the data analysis process. Where verbatim direct quotes are presented, data source of participants is provided for clarity.

All quantitative analyses were performed using R (version 4.3.3; R Core Team 2024) and RStudio (version 2023.12.1.402, release name: “*Ocean Storm*”; RStudio Team 2023) software. Data were visually inspected to identify irregularities or errors, and only participants who provided both baseline and follow up (week 8) accelerometer data were included in the analysis (i.e., a complete case analysis). In accordance with best practices for feasibility and pilot studies [40], statistical analyses were primarily descriptive, focusing on counts, means with standard deviations (SD), or medians with interquartile ranges (IQR). Group comparisons were made using mean differences and 95% Confidence Intervals (CIs). Analyses assessed recruitment, loss to follow-up, participant characteristics, and baseline and follow-up outcome variables.

For quantitative analyses, accelerometers and self-reported data were compared at baseline and at follow-up and was compared between intervention and control groups using analysis of covariance (ANCOVA). A model accounting for clustering (students nested within schools) resulted in an Intraclass Correlation Coefficient (ICC) of 0.00, and an almost identical Akaike Information Criterion (AIC) value compared to our ANCOVA model (AIC = 857 vs. 855).

## Results

### Recruitment and retention

**Fig 1:**
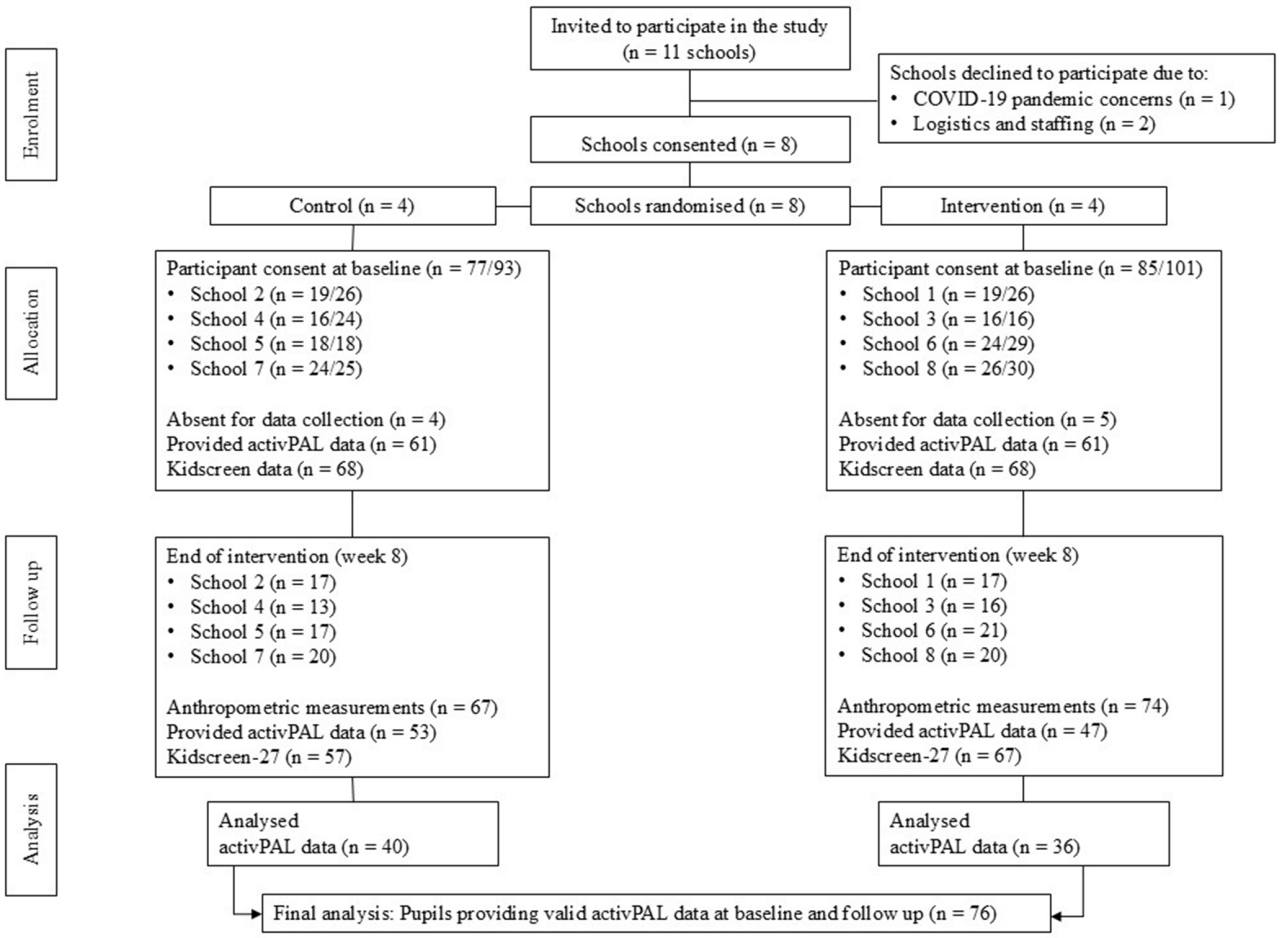
Flow of participants through the *C-SLAMM* intervention (based on CONSORT flow diagram)*. *Only participants that provided both baseline and postintervention data were included within subsequent analyses*.

Fig 1 presents a CONSORT flow diagram of participants through the study. Of the 11 schools initially contacted to take part in the study, two schools declined to participate due to logistics and staffing and one school declined due to COVID-19 concerns. Eight schools agreed to participate. Invitational letters, along with consent forms, were sent to 194 parent/guardians across the eight participating schools. As outlined in Fig 1, a total of 162 parents/guardians and participants returned signed consent and assent forms (84% response rate). The study was conducted in two phases: Phase 1 (n = 70 participants) included four schools (two intervention, two control) and Phase 2 (n = 92 participants) included four additional schools (two intervention, two control). The final sample comprised 162 participants, including 77 girls and 85 boys (Table 2).

**Table 2:** Baseline descriptive characteristics of participants (n = 162).

	Overall	Control	Intervention
*N* schools	8	4	4
*N* (%)	162 (100)	77 (47.5)	85 (52.5)
*N* per school	20 ± 3.9	19 ± 3.4	21 ± 4.6
N (%) per phase:			
• Phase 1	70 (43)	35 (50.0)	35 (50.0)
• Phase 2	92 (57)	42 (45.7)	50 (54.3)
Girls, *N* (%)	77 (47.5)	38 (49.4)	39 (45.9)
Class, *N* (%)			
P4 (7–8 years old)	76 (46.9)	34 (44.7)	42 (55.3)
P5 (8–9 years old)	86 (53.1)	43 (50.0)	43 (50.0)
Height (cm)	134 ± 7.7	135 ± 7.4	133 ± 7.8
Mass (kg)	31.8 ± 8.3	33.2 ± 8.7	30.6 ± 7.8
BMI (m/kg^2^)	17.5 ± 3.0	17.9 ± 3.3	17.2 ± 2.8

P4: Primary 4. P5, Primary 5. BMI: body mass index. *n*, number of participants. Data presented as mean ± SD, unless otherwise specified.

### Qualitative measures

**Fig 2:**
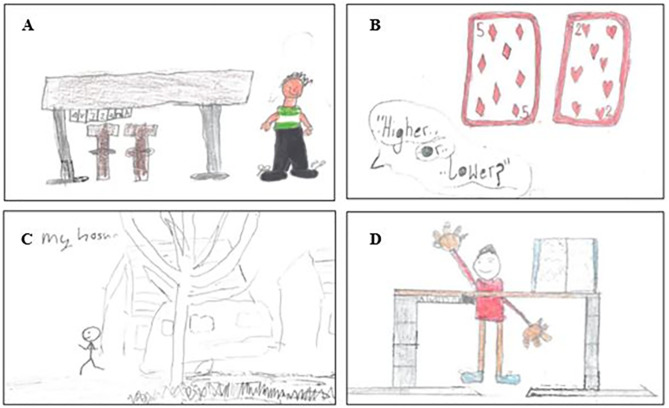
Exemplars of the write and draw activity. A sub-sample of children’s illustrations showcasing their favourite aspects of the *C-SLAMM* intervention, as part of the write and draw activity. **(A)** Drawing from a boy (School 3) demonstrating a sit-stand desk. **(B)** Drawing from a girl (School 2) illustrating a classroom-based card activity. **(C)** Drawing from a girl (School 1) illustrating the active homework component, where she engages in PA at home. **(D)** Drawing from a boy (School 4) illustrating a sit-to-stand desk.

Forty-eight primary-school aged children (25 boys, 23 girls) completed the write and draw activity and focus group discussions. Across the write and draw activity, 203 marks on specific themes were noted, with children illustrating their favourite aspect of the intervention. A sub-sample of children’s illustrations showcasing their favourite aspects of the *C-SLAMM* intervention, is presented in Fig 2. These include sit-stand desks, active homework components and classroom activities.

Teacher interviews were completed by four teachers (80%) from three intervention schools, with one teacher unable to participate due to illness. Teachers reported that the intervention was straightforward and easy to implement within daily classroom practices and found the intervention components appropriate for their classes. Four themes emerged from all three data sources (e.g., write and draw, focus groups and teacher interviews): (1) engagement, (2) positive aspects of *C-SLAMM* intervention, (3) barriers to *C-SLAMM* intervention delivery, and (4) recommendations for improvement. These themes were further divided into specific sub-themes (see Table 3).

**Table 3:** Themes and sub-themes identified from qualitative data analysis.

Themes	Sub-theme
**Engagement**	Fun and play Enjoyment Teachers’ confidence in the delivery of resources
**Positive aspects of *C-SLAMM* intervention**	Increased range of physical activity opportunities Educational Increased competence Increased choice and flexibility
**Barriers to *C-SLAMM* intervention delivery**	Parental support Lack of classroom space Allocation of sit-to-stand desks Lack of time
**Recommendations for improvement**	Wider engagement Collaborative knowledge sharing Environmental changes Expand on the *C-SLAMM* intervention

Qualitative data sources include children’s write and draw activity and focus group discussions and teacher interviews. *C-SLAMM* = Children- Sit Less, Move More.

### Engagement

During focus group discussions, children consistently described the *C-SLAMM* intervention as “*fun*” (n = 24). When children were asked to write their favourite component of the intervention, children frequently wrote words such as “*move more”*, “*play”* and “*fun”.* The most common illustrations were of sit-stand desks, games and outdoor activities, either during lunch breaks or at home. This was consistent across all six focus groups. Teachers also noticed children’s positive reactions, explaining that pupils looked forward to health lessons, active homework and breaks from sitting. One teacher explained: “*it breaks the monotony…there’s something to look forward to, that bit of excitement, what lesson are we going to do this time? Is there something we like, is there something that we know, is something different*?” (Teacher, School 4, Interview data).

Furthermore, teachers viewed the teacher training and *C-SLAMM* intervention manual positively. One teacher stated the intervention would be “*very beneficial for the school”* and that the *“resources were brilliant*” (Teacher, School 1, Interview data). All teachers praised the “*minimal*” workload. Teachers found the resources clear, easy to implement, and noted that teachers did not have to come from a sporting background to implement the resources. One teacher commented that the “*file is very well laid out…you can really sit down and take all the main things from it*” (Teacher, School 4, Interview data). Teachers recognised the importance of the manual, which they used to refresh their knowledge of the activities and the key messages to deliver. One teacher shared: “…*teachers would welcome it….it [C-SLAMM intervention resources] really makes your teaching much, much better*” (Teacher, School 2, Interview data).

### Positive aspects of *C-SLAMM* intervention

Children and teachers were generally positive about the *C-SLAMM* intervention (S5 File, Table 1). During focus group discussions, children highlighted the importance of making the intervention available across the entire school as “*it would be more like fair instead of one class in one school they get it and they would have to do all these fun stuffs and the other classes wouldn’t”* (Child, School 2, focus group data).

Both children and teachers appreciated the variety of methods used to deliver breaks and health lessons, highlighting that some children naturally preferred standing. Teachers noted that the variety of *C-SLAMM* components – such as health lessons, active homework, and breaks from sitting – could be easily adapted and incorporated with minimal additional workload. Several teachers described the positive impact *C-SLAMM* intervention had on their classroom teaching experience. One teacher reflected “*I just love the lesson….it really did educate children about being active and about being sedentary”*(School 4, interview data). Pedometers, which were consistently used across intervention schools, facilitated movement during lunchtime and supported other implement intervention components (e.g., active breaks and active lessons). Children enjoyed using the pedometers as a tool for tracking their PA. One teacher shared that the children “*…asked for them every single day*” (Teacher, School 2, interview data).

Positive behaviour was observed when regular breaks from sitting were integrated throughout the school day. One teacher highlighted the engagement and improved demeanour of children, particularly those with anxiety and autism: “*I’ve noticed the children love them (sit-stand desks) and they’re smiling and it’s fun. The wee ones with anxiety are loving it… even one boy, he would have quite severe autism, he doesn’t really leave his seat, but when we were doing some of the active breaks, I would say, ‘move somewhere else in the room’…. he actually started to move from his chair but before this he would’ve never moved from his chair*” (Teacher, School 1, Interview data).

### Barriers to *C-SLAMM* intervention delivery

Four sub-themes relating to barriers to *C-SLAMM* intervention delivery were identified by teachers: insufficient parental support, limited classroom space, allocation of sit-stand desks and lack of preparation time.

Teachers faced challenges with facilitating active homework due to insufficient home support and feedback from parents and guardians. One teacher reflected, “*you’re always reliant on that one or two good parents, my father always told me that one good parent is worth three teachers… I always knew that the parents have to be onboard as well to push that too”* (Teacher, School 4, Interview data). The challenges were worsened by the COVID-19 pandemic, which further reduced direct communication with families. One teacher remarked, “*With parent support and with two lockdowns, we’re finding it overall very hard to get homework back…if it goes home, we want the message to be replicated or repeated by parents again, just to drive it home… You know, they come to the gate and then there away”* (Teacher, School 1, interview data).

All teachers stated that classroom size and space limitations made storage and material management difficult. One noted, “*unless there was a trolley or something with trays or shelfing, I felt lost for that individual space the children normally have”* (Teacher, School 4, Interview data). Ensuring partial allocation of sit-stand desks was challenging, with teachers citing logistical and scheduling issues. One teacher stated, “*we didn’t get enough time to figure out a system… because some kids work best standing, but it was hard to get a system put in place…half class full of desks and half not”* (Teacher, School 1, Interview data). Another added, “*it was great whenever we collectively stood at the desks, but whenever it was down to the children’s choice, both parties had to agree. We’re standing, we’re sitting”* (Teacher, School 2, Interview data). In addition, teachers stated that they needed more time to integrate materials effectively. One reflected, “*a little bit of time to get to know the material, to absorb the material to know, ah, there’s an active lesson I think I can link into this”* (Teacher, School 1, Interview data).

### Recommendations for improvement

Overall, all focus groups (children) and semi-structured interviews (teachers), deemed the *C-SLAMM* intervention to be an acceptable programme that encouraged PA in school and at home. Both children and teachers interviewed supported expanding the *C-SLAMM* intervention to schools across NI. One teacher stated, “*I would have no hesitation in saying to the other classes, right here’s a bank of resources, great ideas, implement them*” (Teacher, School 4, Interview data). Focus group and interview data highlighted specific feedback regarding the allocation and distribution of sit-stand desks. Both children and teachers expressed a clear preference for individual desks (e.g., one desk per child) and additional under-desk storage.

Teachers highlighted the importance of parental involvement and collaborative efforts to ensure the intervention’s success. They advocated for greater autonomy in delivering the programme and stressed the need for sufficient time and resources. To enhance the adoption and implementation of the intervention, teachers recommended several strategies: wider engagement, collaborative knowledge sharing, environmental changes and expanding the *C-SLAMM* intervention (see S5 File, Table 2). Teachers advocated for increased dissemination of online resource for parents, hosting information sessions for guardians, fostering collaborative knowledge sharing, and adapting the learning environment to better suit programme goals. Teachers supported recommendations for improving teacher training and the *C-SLAMM* intervention manual included the development of an online database or portal featuring videos from various schools. This resource would demonstrate different active breaks, active lessons, and active homework strategies. Importantly, all teachers expressed strong support for implementing the *C-SLAMM* intervention in their own schools and throughout NI.

Importantly, all teachers expressed strong support for implementing the intervention in their own schools and throughout NI. They recognised the potential to improve health and enhance learning environments. As one teacher stated, “…*health and exercise and the brain working in shorter chunks and more productivity from the children in terms of what to do…if people buy into that concept, which I do think teachers will, I don’t see why this couldn’t be rolled out or why this couldn’t take a greater role within Northern Ireland”*(Teacher, School 1, interview data).

### Teacher logbook and fidelity checks

Teachers from three of the four intervention schools completed weekly logbooks, with a researcher (SN) conducting weekly fidelity checklist in these schools. In the fourth school, the intervention was not delivered during the final three weeks (Weeks 5–8) due to teacher illness, resulting in only 30% of the fidelity checklist being completed. Communication between the teacher and the researcher (SN) was limited during this period, resulting in the absence of a post-intervention interview.

Although all intervention teachers attempted to complete the logbooks, due to extensive missing entries, we were unable to use the data. Logbooks highlighted differences in teacher “*buy-in”* and engagement. For example, one teacher developed complementary materials, such as *C-SLAMM* intervention booklets and wall art, and allocated more time to the intervention than others. Due to the varying nature of the logbooks, it was not possible to quantify adherence and exposure to the intervention.

### Child-level outcomes

#### Sitting and physical activity.

**Fig 3:**
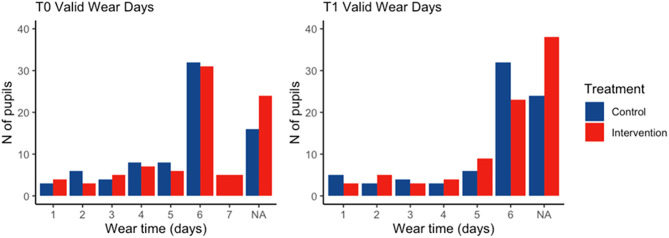
ActivPAL wear time compliance at baseline (T0) and follow-up (T1) for the study cohort (n = 162).

Of the 162 participants, 115 (94.3%) had sufficient wear time (≥2 days of accelerometer data) at baseline, and 92 (92%) had sufficient wear time at follow-up. ActivPAL wear time compliance at baseline (T0) and follow-up (T1) for the study cohort is presented in Fig 3.

The median (IQR) number of valid wear days in the intervention group was 6 (2) at both baseline and follow-up. In the control group, the median (IQR) was 6 (2) valid wear days at baseline and 5 (2) at follow-up. Just over half of the children had six valid wear days at baseline (intervention vs. control: 50.8% vs. 52.5%; N = 31 vs. 32) and at follow-up (48.9% vs. 60.4%; N = 23 vs. 32). The median (IQR) wear time for the intervention group was 8,640 (2,880) minutes at baseline and 7,200 (2,880) minutes at follow-up, while for the control group, the median wear time remained consistent at 8,640 (2,880) minutes for both time points.

Only 76 participants (46.9%) met the wear time criteria at both baseline and follow-up, with 40 (53%) participants in the control group and 36 (47%) in the intervention group (Table 4). At baseline, nearly a quarter of participating children (24.7%; n = 40) had missing accelerometer data, and this proportion increased to 38.3% (n = 62) at the 8-week follow-up. Missing data were more prevalent in the intervention group compared to the control group at both baseline (28.2% vs. 20.8%; n = 24 vs. 16) and follow-up (44.7% vs. 31.2%; n = 38 vs. 24). The extent of missing data varied across schools, with the proportion of children with valid data ranging from 32% to 78% depending on the school. Given the discrepancies in missing data between treatment groups and across schools, the data were determined to be not missing at random. As a result, data imputation was deemed inappropriate. ActivPAL wear time compliance at baseline and follow-up assessments for the study cohort (n = 162) is presented in S4 File.

**Table 4:** Between-group comparison for activPAL data at baseline and follow-up (week 8).

	All (n = 76)	Control (n = 40)		Intervention (n = 36)		Intervention vs. control (n = 76)
	Baseline	Baseline	Follow-up	Baseline	Follow-up	MD* (95% CI)
Time sitting (mins/day)	363 ± 73	356 ± 83	344 ± 69	371 ± 61	345 ± 82	−6.5 (−36.4, 23.4)
Sit-to-stand transitions (N/day)†	95 (81, 117)	93 (80, 112)	99 (82, 114)	100 (83, 122)	96 (81, 105)	−6.8 (−16.4, 2.8)
Time standing (mins/day)	175 ± 48	173 ± 50	179 ± 43	178 ± 47	178 ± 47	−2.9 (−22.2, 16.5)
Step count (N/day)	10,034 ± 2,564	9,975 ± 2,387	10,735 ± 3,329	10,100 ± 2,781	10,390 ± 3,190	−409 (−1,783, 966)
Time stepping (mins/day)	130 ± 32	130 ± 30	139 ± 40	130 ± 35	132 ± 36	−7.2 (−23.2, 8.8)
METs	34 (32, 35)	34 (32, 35)	33 (31, 35)	34 (32, 35)	31 (29, 34)	−1.7 (−3.3, −0.1)
**School time data** ^a^						
Time sitting (mins/day)	195 ± 43	191 ± 46	192 ± 40	199 ± 38	188 ± 35	−7.2 (−22.6, 8.3)
Sit-to-stand transitions (N/day)	48 ± 18	48 ± 19	47 ± 16	47 ± 17	46 ± 13	−0.8 (−6.1, 4.4)
Time standing (mins/day) †	88 (70, 111)	94 (75, 108)	96 (77, 110)	84 (68, 113)	88 (77, 121)	1.7 (−10.7, 14.1)
Step count (N/day)	5,447 ± 1,319	5,350 ± 1,496	5,513 ± 1,810	5,555 ± 1,102	5,855 ± 1,185	223 (−398, 843)
Time stepping (mins/day)	69 ± 16	69 ± 17	70 ± 21	69 ± 14	73 ± 14	3.2 (−4.1, 10.5)
METs	9.9 (9.5, 10.2)	9.8 (9.5, 10.1)	10.1 (9.2, 10.3)	9.9 (9.6, 10.2)	10.0 (9.8, 10.4)	0.1 (−0.2, 0.4)

^a^School-time filter (09:00–15:00); *n*, number of. mins, minutes. This table includes data from participants who met the minimum wear time criteria of 10 waking hours for ≥ two days at baseline and at follow-up (control *n* = 40, intervention *n* = 36). Descriptive data presented as mean ± SD or median (quartile 1, quartile 3). *Mean Difference (MD) between groups are expressed via baseline adjusted mean differences (95% Confidence Intervals).

Table 4 provides group comparisons of activPAL variables recorded during waking hours and school time (09:00–15:00) for the control and intervention groups at baseline and follow-up (Week 8). At baseline, children (n = 122) spent an over 6 hours (363 ± 80 minutes) sitting per day. During an average school day, children (n = 122) spend more than half of their time sitting (195 ± 43 minutes). At follow-up, there were no differences between the control and intervention groups in terms of time spent sitting, standing, stepping during the school day.

#### Challenges with accelerometery measurement.

Both children and teachers identified challenges associated with activPAL monitors. During focus group discussions, children in all focus groups reported discomfort while wearing the devices, citing issues such as skin irritation, marks left on leg by the stickies, and interference with sport activities. One child shared, “*it kept rubbing… and then I got a rash when I took it off*” (Child, School 2, Focus Group Data). Another stated, “*I didn’t really like it cause when you’re taking it off, it feels painful*.” (Child, School 1, Focus Group Data). Additionally, some children found the monitors distracting: “*it’s like a little bit annoying because when you wear it, like itchy a lot and then it hurts when you try to itch it*” (Child, School 4, Focus Group Data).

### Health-Related Quality of Life

There was no differences in Kidscreen-27 responses over time (considered as T-scores) between the intervention and control group indicating no impact of intervention on HRQoL (see S5 File).

## Discussion

This study aimed to assess the feasibility and acceptability of implementing the multicomponent intervention (*C-SLAMM*), adapted from the efficacious Australian *TransformUs* programme to reduce sitting time and increase PA in P4 and P5 school children (aged 7–9 years) in NI. While elements of the *TransformUs* intervention informed the *CLASS-PAL* intervention implemented in England [60], this is the first known multicomponent school- and home-based intervention adapted from the *TransformUs* programme, to attempt to improve PA and reduce sitting time in primary school children (aged 7–9 years) to be undertaken in NI.

The study specifically sought to assess the feasibility of recruiting and retaining children, evaluate the appropriateness of data collection procedures, determine the acceptability of intervention implementation and explore preliminary effectiveness on children’s PA and SB levels. Qualitative findings from the write and draw activity, focus groups and semi-structured interviews revealed that the content of the *C-SLAMM* intervention was engaging and suitable for the Northern Irish context. However, some suggestions on delivery modifications were highlighted. These included increasing the number of sit-stand desks allocated to each class (e.g., one desk per child) with additional under-desk storage, engaging the entire school community in the intervention, fostering collaborative knowledge sharing and increasing parental involvement.

Of note were the findings that all participating schools completed the study, and the recruitment strategy was deemed successful as the response rates were high (84%). The high recruitment rates for the *C-SLAMM* intervention could be due to friend/peer involvement or support [61], due to small rewards or incentives (e.g., a pencil) [62] or children may have perceived the intervention to be fun [63]. Previous school-based PA interventions have examined possible motivating factors to school-based PA intervention participation and state that effective recruitment strategies contribute to the success of PA interventions [62,64,65]. Overall, the recruitment and retention methodology used in the *C-SLAMM* intervention were positively received, with 87% of primary-school children (aged 7–9 years) retained at follow-up (Week 8). Due to variation in sample sizes, it is difficult to directly compare retention rates between intervention studies. However, the *C-SLAMM* intervention demonstrated higher retention rates than a similar study conducted by McLellan *et al.* [66] which reported an 80% retention rate And the *TransformUs* intervention, in which 24% of children dropped out or were unavailable to participate in data collection [31].

Qualitative results revealed that both children and their teachers considered the *C-SLAMM* intervention to be a positive, fun, and acceptable programme in school, adding variation, fun and enjoyment to the school and home-setting. This finding is in line with previous research, suggesting that PA can increase children’s enjoyment and engagement at school [29,67]. Combining the visual and verbatim data enhanced data credibility, and revealed findings on children and teachers’ views, experiences and perceptions of the *C-SLAMM* intervention which were not captured in the quantitative data. For example, teachers identified the inclusion of breaks from sitting into the subject curricula as a facilitator.

Teachers reported that children’s enjoyment was a key factor for influencing their willingness to continue integrating breaks from sitting into the classroom. Children provided positive feedback regarding the *C-SLAMM* intervention, describing improvements in their ability to concentrate and feel better (S3 File). Furthermore, the intervention improved teachers’ confidence and willingness to integrate active pedagogies into current and future teaching practice with all the teachers interviewed indicating a willingness to implement strategies. Hence, given that child enjoyment has frequently been recognised as an important factor when considering teacher perceptions of additional movement in the classroom [68,69] and acts as a key facilitator to implementation of PA interventions [70], this is something that should continue to be considered with respect to movement integration interventions.

At baseline, the findings of the current study indicate that primary school children (aged 7–9 years) in NI spend over 6 hours of their typical day sitting and around 54% of their school-day sitting. The high level of sitting time observed in the *C-SLAMM* intervention is a serious public health concern, given the links with cardiometabolic risk, fitness and body composition in children [3,71]. The results concur with previous research conducted in the UK, that found children (*n* = 243, aged 10–11 years) spend large portions (up to 69%) at school in sedentary time [72] and approximately 70% of their school-day sitting (*n* = 30, aged 9–10 years) [35]. Our baseline data collection took place in Autumn 2021, when lockdown restrictions had mostly been removed. The effects of the COVID-19 pandemic and subsequent restrictions on habitual PA levels, may have contributed to the high levels of sitting time observed [73]. The impact of the COVID-19 pandemic in children, particularly how movement behaviours (e.g., PA and SB) have changed over time are still yet to be understood [74,75].

One intervention teacher failed to complete their logbook, fidelity checklists and semi-structured interview, limiting the evaluation of intervention fidelity and acceptability. Teacher logbooks indicated that some components of the intervention (such as breaks from sitting or active lessons) may not have been implemented as intended potentially contributing to the absence of a significant effect on sit-to-stand transitions. Similar to findings from the *TransformUs* intervention, where teacher diary response rates varied widely across sites (ranging from 37% to 74%) [76], variability in implementation fidelity was observed in this study. The components most consistently implemented were most likely those perceived as easiest to integrate within the existing school routine [62]. Factors affecting implementation (e.g., perceived barriers and facilitators) are often evaluated after the intervention is conducted, and the feasibility of introducing a school- and home-based PA intervention prior to implementation is not always reported [29]. Understanding these factors is essential to interpreting the success or lack of success of an intervention [29,77]. In line with findings of previous research, the successful implementation of *C-SLAMM* intervention was influenced by a variety of factors (e.g., time constraints, lack of classroom space and availability of resources) [67,69,77]. Qualitative findings also revealed that substitute teachers were employed in all intervention schools during the 8-week intervention period due to teacher absences, and one school did not implement all intervention components, reducing potential transferability of the results. Therefore, prior to commencing a fully powered cRCT, investigators should consider incorporating future fidelity checks (e.g., observation, using accelerometer data or questionnaires) to ensure that breaks from sitting occur every 30 minutes (as per *C-SLAMM* intervention protocol) [78].

Over the eight-week intervention period, no significant changes were observed between intervention and control groups for sitting time. Although PA and sitting time were robustly measured using established methods [49,52], high levels of missing data limited the sample size for which PA and sitting time could be analysed. The inclusion of accelerometer- based outcomes aimed to determine whether the *C-SLAMM* intervention influenced movement behaviours. However, if activPAL determined sitting time were retained in a fully powered cRCT, further modifications to the wear protocol are warranted to ensure improvements to compliance rates. Hip-worn accelerometers may pose challenges in younger populations, with children reporting discomfort and skin irritation, issues that are consistent with previous research in children and adolescents [78,79]. Nevertheless, they remain the most accurate method for detecting postural changes, highlighting the need to balance acceptability with measurement precision [80]. Findings from semi-structured focus groups with children and one-to-one interviews with teachers, highlighted challenges with research measures, particularly that children experienced skin irritation from the medical dressing(s), leading to the refusal to wear the device. Similar issues were observed in the ‘*Stand Out In Class’* pilot cRCT, which aimed to increase children’s PA and decrease sitting time using sit-stand desks [78,81]. Unlike the *C-SLAMM* intervention, the *Stand Out in Class* study used both activPAL and ActiGraph devices to measure children’s sitting time and reported that lower compliance rate for the activPAL compared to the ActiGraph (63% *vs* 83%) [78,81].

Challenges associated with activPAL compliance may also reflect broader methodological limitations. Currently, there is no gold standard for processing and analysing activPAL data, and the data reduction methods are subject to researcher bias when excluding periods of non-wear or isolating periods of interest [82]. Additionally, while activPAL software exports daily stepping cadences ≥ 100 steps per minute (spm), a widely recognised threshold for MVPA, this threshold varies based on individual factors such as BMI, potentially leading to misclassification of activity intensities [83]. Thus, future work should consider the feasibility of including both activPAL and other accelerometer devices, as accelerometer software can estimate time spent inactive and the activPAL software can accurately measure sitting, standing, walking and postural transitions in children [49,84,85].

Qualitative results highlighted that the *C-SLAMM* intervention was acceptable and strongly supported by teachers and children, with participating schools citing positive aspects such as the increased range of PA opportunities. Nevertheless, several factors may explain the lack of a significant intervention effect. One possible reason may be the length of exposure, as the intervention ran for an 8-week period, which may not have been sufficient to elicit improvements in PA [86,87]. A systematic review investigating school-based PA interventions in children and adolescents (aged 6–18 years) found that time is an essential variable for changes to occur, with interventions with a duration of less than three months presenting fewer effects than those of a longer duration (e.g., 3 months to 1 year) [88]. Environmental factors, such as changes in local PA and school sport policies, were also not considered. Another possible explanation is the high baseline levels of PA in the control group, a common issue in school-based PA interventions [89]. In addition, the small sample size may have limited the ability to detect meaningful changes in sitting time. However, it is worth noting that as a feasibility pilot, this study was not designed or powered to detect intervention effects but rather to assess implementation. Thus, conducting a retrospective sample size calculation is inappropriate, as the study was not originally powered for this analysis [90]. In contrast, the *TransformUs* cRCT, conducted in over 300 Australian students, effectively reduced children’s SB [31] and found that accumulating light-intensity PA in shorter bursts were more favourable than longer bouts of the same intensity, for reducing BMI [33]. Importantly, prior to evaluating the *C-SLAMM* intervention for effectiveness, the authors sought to assess the acceptability and feasibility to identify potential issues with compliance, delivery of the intervention, recruitment and retention rates [42,91].

Similar to previous studies [18], this study demonstrates that the context, specifically the school setting, the facilities and skill of school staff as well as school size and ethos, played an integral role in the implementation of the intervention. All teachers interviewed stated that they will implement the programme after the intervention. Teachers noted that further dissemination of information was warranted (e.g., an information event, online communications) to improve engagement with parents. Overall, teachers indicated they would like to have received some evaluation from parents/guardians regarding active homework and the newsletters. Pre-planning, preparation time and a period to allow teachers to familiarise themselves with the materials ahead of starting the intervention is critical. Previous school-based PA found that a ‘*whole school approach’* is feasible to promote PA in primary-schools [92]. In line with these findings, teachers felt that wider cultural awareness of the intervention was required across the school- and home-setting, to aid intervention sustainability.

### Strengths and limitations

The mixed-methods approach involving multiple data sources, provided valuable insights into the feasibility and acceptability of the *C-SLAMM* intervention from the perspectives of both children and teachers. By combining device-based quantitative measurements of sitting time and PA with personal accounts, the study captured a deeper understanding of the meaning behind children’s PA behaviours. The study incorporated diverse data sources, including perspectives from children from four schools across NI. Methodological strengths include the exploration of consensus and associated discussion through focus groups. Perspectives from children via write and draw activity/focus groups, teachers via interviews, helped to provide a comprehensive picture of the intervention and how it was received and implemented. This study contributes to the expanding literature of multicomponent school- and home-based PA interventions. The intervention represents an adaption of the evidence-based *TransformUs* programme, tailored to the NI setting. To our knowledge, no similar multi-component programme has been subjected to rigorous feasibility and pilot testing within the NI/UK context. A key strength of the *C-SLAMM* intervention is that it was adapted through formative work and a development phase, using a co-design process to ensure that its components were appropriate for primary schools in NI. This process actively engaged parents and teachers of primary schoolchildren, whose perceptions, views, and experiences regarding classroom, school- and home-based PA were incorporated into the intervention’s development [37]. The intervention was low-cost and required few resources to implement within the school- and home-setting. A key strength to this study was the in-person teacher training, which was conducted ahead of the intervention.

While this study confirms important insights into perceived likes and dislikes of the *C-SLAMM* intervention among both primary-school children (aged 7–9 years) and their teachers, parental feedback is lacking. Thus, conclusions on the effectiveness of this intervention in the home and home setting may be limited. Additionally, the intervention is limited due to its short intervention period (8 weeks) and the lack of long-term follow-up. Environmental factors were not considered, and detailed demographic data on participating schools were not collected, which may limit the generalisability of the findings. Moreover, inconsistent delivery logs and the absence of a teacher interview from one school prevented assessment of how many components were delivered and implemented. Potential inequalities in PA outcomes (e.g., sex differences or variations between more and less active children) were not explored and children and teachers may not have been completely honest when discussing certain aspects of the intervention, e.g., compliance when wearing the activPAL monitor (versus actual wear time), due to social desirability bias [93]. These limitations should be addressed in future research to strengthen the design, delivery and evaluation of multi-component interventions in primary-school settings.

## Conclusions

The study was well-received by both children and teachers, highlighting its potential as an acceptable strategy for promoting PA. The observed variations in intervention dose and implementation fidelity highlight the challenges associated with intervention delivery within school settings, suggesting that a flexible design may be necessary [34]. Due to inadequate intervention implementation of the intervention and missing accelerometer data for some participants, definitive conclusions regarding the impact of the intervention on children’s sitting time and PA levels could not be drawn. Nevertheless, intervention components were welcomed, with children and teachers finding them an acceptable method for encouraging primary-school children (aged 7–9 years) to “*sit less and move more*”. Barriers identified by children and teachers, in relation to time, lack of parental support and lack of classroom space should be addressed. Positive experiences and recommendations identified by teachers in this study offer valuable insights for the implementation of similar interventions in real-world settings, particularly within the NI educational system. These insights could inform the future adaptation and scaling of school- and home-based SB and PA interventions. Incorporating strategies such as breaks from sitting, health lessons and environmental changes to promote PA and reduce sitting time, may be effective in promoting PA during school time. Additionally, findings also suggest the need to consider alternative evaluation designs to account for contextual differences and varied deliveries of an intervention across multiple schools. It is essential that the *C-SLAMM* intervention considers the context of each participating schools as failure to do so would affect generalisability and scalability of the programme. Although participants were enthusiastic about the intervention, further refinement of outcome measures are warranted before advancing to a fully powered cRCT.

## Supporting information

S1 File Flow diagram for the Children – Sit Less Move More (C-SLAMM) intervention. (DOCX)

S2 File Summary of the Children – Sit Less, Move More (C-SLAMM) intervention outcome measures. (DOCX)

S3 File Qualitative measures. A summary of the Write and Draw activity, the focus group topic guide for children, and the semi-structured one-to-one interview guide for teachers.
(DOCX)

S4 File Qualitative findings. Presents quotations from children and teachers highlighting perceived positive aspects of the intervention, as well as teacher recommendations for improving the *C-SLAMM* intervention.
(DOCX)

S5 File Child-level outcome measures. Includes a table summarising ActivPAL wear time compliance at baseline and follow-up assessments for the study cohort, as well as a table presenting the Kidscreen-27 results (Median, IQR) comparing the *C-SLAMM* intervention and control groups.
(DOCX)

S6 File CONSORT C-SLAMM. (DOCX)

S7 File Study protocol for ethics. (PDF)
