# Supplementary material for: The Children – Sit Less, Move More (C-SLAMM) pilot intervention: Feasibility and acceptability of a multi-component school and home-based intervention to promote physical activity
Source: PLoS One. 2025 Nov 19;20(11):e0335933. doi: 10.1371/journal.pone.0335933 (PMC12629496; doi:10.1371/journal.pone.0335933)
Supplement: S1 File — (DOCX) [file pone.0335933.s001.docx]

**Supplementary File 1.** Flow diagram for the *Children - Sit Less, Move More* (C-SLAMM) intervention
