# Supplementary material for: The Children – Sit Less, Move More (C-SLAMM) pilot intervention: Feasibility and acceptability of a multi-component school and home-based intervention to promote physical activity
Source: PLoS One. 2025 Nov 19;20(11):e0335933. doi: 10.1371/journal.pone.0335933 (PMC12629496; doi:10.1371/journal.pone.0335933)
Supplement: S2 File — (DOCX) [file pone.0335933.s002.docx]

**Supplementary File 2.** Summary of the *Children – Sit Less, Move More (C-SLAMM)* intervention outcome measures.

| **Feasibility outcome measure** | **When administered** | **Participants** | **Mode of response** |
| --- | --- | --- | --- |
| Recruitment via consent forms | Before baseline measurements | Children + classroom teachers | Signed informed consent |
| Logbook | Weekly | Classroom teachers | Open reflection diary |
| Fidelity checklist | Weekly | Classroom teachers | Tick the box checklist |
| Sitting and PA outcomes | Baseline and follow-up | Children | ActivPAL |
| Anthropometrics | Baseline and follow-up | Children | As per protocol for measure (see Anthropometrics section) |
| HRQoL | Baseline and follow-up | Children | Kidscreen-27 questionnaire, 5-point Likert scales |
| Focus group and write and draw activity | After the intervention was completed (week 9) | Two groups of six children (12 children per school) | Write and draw task to express their perceptions and experiences of the intervention components visually and independently. Oral responses to open-ended questions |
| Semi-structured interviews | After the intervention was completed (week 9) | Classroom teachers | Oral responses to open-ended questions |

PA = physical activity; HRQoL = Children’s health-related quality of life
