# Supplementary material for: The Children – Sit Less, Move More (C-SLAMM) pilot intervention: Feasibility and acceptability of a multi-component school and home-based intervention to promote physical activity
Source: PLoS One. 2025 Nov 19;20(11):e0335933. doi: 10.1371/journal.pone.0335933 (PMC12629496; doi:10.1371/journal.pone.0335933)
Supplement: S3 File — A summary of the Write and Draw activity, the focus group topic guide for children, and the semi-structured one-to-one interview guide for teachers. (DOCX) [file pone.0335933.s003.docx]

**Supplementary File 3.** Write and draw activity and focus group guide

**
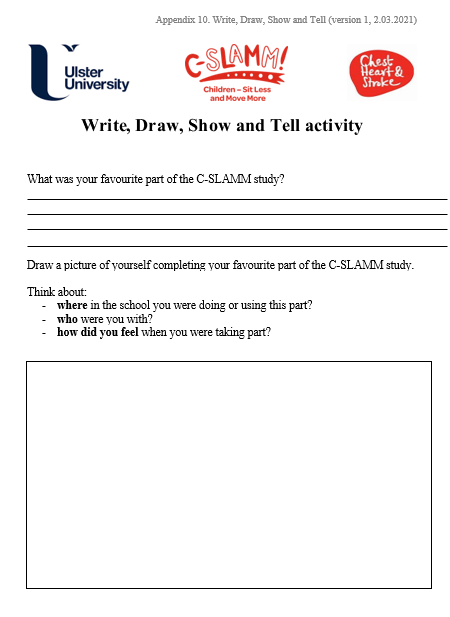
**


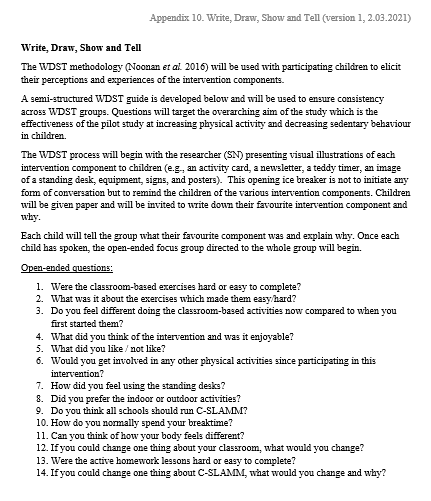


**Semi-structured topic guide for Teachers (Interviews)**

1. What has been your favourite and least favourite component of the C-SLAMM study?
2. What were the strengthens and weaknesses of the teacher training?
3. Do you have any suggestions and/or ideas that will assist us in future teacher trainings in this topic?
4. During the eight-week study, what components of the study were the easiest to deliver to the class? Conversely, what components were more challenging to deliver/maintain to the class?
5. What has been the biggest problems, barriers, or challenges for you as a teacher involved in the C-SLAMM study?
6. In relation to the equipment package, what equipment has been most used and what equipment has not been used? Is there any additional equipment, that has not been included in the study, that could be used in further work?
7. If you could change anything about the study, what would you change and why?
8. Do you have any further recommendations or ideas that could be used in a physical activity intervention in P4/P5 children?
9. Is there any components or techniques within in the C-SLAMM study that you will continue to use in your classroom/school?
10. How practical do you think it is to run this permanently the school?
11. Would you consider it feasible to roll out this intervention to all schools?
