# Supplementary material for: The Children – Sit Less, Move More (C-SLAMM) pilot intervention: Feasibility and acceptability of a multi-component school and home-based intervention to promote physical activity
Source: PLoS One. 2025 Nov 19;20(11):e0335933. doi: 10.1371/journal.pone.0335933 (PMC12629496; doi:10.1371/journal.pone.0335933)
Supplement: S4 File — Presents quotations from children and teachers highlighting perceived positive aspects of the intervention, as well as teacher recommendations for improving the C-SLAMM intervention. (DOCX) [file pone.0335933.s004.docx]

**Supplementary File 4. Qualitative Findings**

**Table 1.** Data from children and teachers regarding positive aspects of the *Children - Sit Less, Move More* (C-SLAMM) intervention

| **Positive aspects** | **Quotes** |
| --- | --- |
| **Increased range of PA opportunities** | …*definitely do more activities than what we normally do… doing more exciting things than just like boring work.* (Child, School 2, focus group data)  *…main strengths would be the variety of breaks that you have introduced there…the active lesson you've covered so much that can be integrated in with English or history or maths it’s really very, very good teaching the way you've outlined this*. (Teacher, School 3, interview data)  *Getting them [the children] so active, being more conscious of being active in like three/four areas a day, the practical kind of hands-on approach to everything, just getting them out of their seats…They love that. They did just really thrive from that*. (Teacher, School 1, interview data) |
| **Educational** | *I liked when we learned more about standing up than sitting down. Cause when you sit down, you don’t get that much exercise than when you stand up…* (Child, School 1, focus group data)  *I just love the layout of the lesson and it really did explain to the children about being active and about being sedentary… I just enjoyed delivering those lessons. I know there was only five but enjoyed delivering those.* (Teacher, School 1, interview data) |
| **Increased competence** | *Made me concentrate and feel better…* (Child, School 3, write and draw data)  *Those small bursts of learning, you had the children that they weren't exhausted...it wasn't a long monotonous lesson. It was a lesson that was deliberately structured in terms of it breaks so they were more receptive…there's less time looking out the window.* (Teacher, School 1, interview data) |
| **Increased choice and flexibility** | *Throwing and catch it's fun…I liked everything I want to do it again…. my legs sometimes just wanted to be jumpy, so I like the standing up mode cause I really like standing... it makes me move more.* (Child, School 4, focus group data)  *They loved taking part in all of the different things and different resources…they didn't want to miss out on their break or their pedometer challenge or anything like that…the kids really got involved.* (Teacher, School 2, interview data) |

PA = physical activity.

| **Table 2.** Teacher recommendations for improvement of the *Children - Sit Less, Move More* (C-SLAMM) intervention | |
| --- | --- |
| **Recommendations for improvement** | **Quotes** |
| **Wider engagement** | *parents with a little bit of background they too, could have been given the message that I was trying to give in class… so maybe I would say that anything that went home in relation to maybe those newsletters or maybe the part of the health lessons….maybe they could have went home and maybe they could have went a little bit better in terms of more knowledge from the parent so they two could emphasise or they could go over the key points that I was trying to do. So maybe just a wee bit more communication would’ve made all of the tasks, easy to implement* (School 2, interview data)  … *parents could have been given the message that I was trying to give in class…more knowledge from the parent so they too could emphasize, or they could go over the key points that I was trying to do. So maybe just a bit more communication would’ve made all the tasks, easy to implement*. (School 4, interview data) |
| **Collaborative knowledge sharing** | *I would've had my own ideas [to integrate the movement in the class], but it was nice to have that bank to go too and say, oh right someone has went to the effort, thought of an idea, I haven't thought of it, I can see how it can be used.* (School 2, interview data) |
| **Environmental changes** | *one [sit-to-stand desk] for everybody would be beneficial, because that's the hardest bit we found is making sure that they all had a turn and, and using them in, in a way that was fair for them to use it as well* (School 1, interview data) |
| **Expand *C-SLAMM* intervention** | *…a portal whereby you could upload or promote or show one class doing something and if you upload it to show what they’re doing as part of C-SLAMM, one idea could be developed by another class… all the different ideas that you can get from different teachers like a forum to promote C-SLAMM… the more ideas from different schools you have will be better. So that sharing of knowledge and expertise from different teachers, is something that I would try to encourage*. (School 4, interview data) |
