# Supplementary material for: The Children – Sit Less, Move More (C-SLAMM) pilot intervention: Feasibility and acceptability of a multi-component school and home-based intervention to promote physical activity
Source: PLoS One. 2025 Nov 19;20(11):e0335933. doi: 10.1371/journal.pone.0335933 (PMC12629496; doi:10.1371/journal.pone.0335933)
Supplement: S5 File — Includes a table summarising ActivPAL wear time compliance at baseline and follow-up assessments for the study cohort, as well as a table presenting the Kidscreen-27 results (Median, IQR) comparing the C-SLAMM intervention and control groups. (DOCX) [file pone.0335933.s005.docx]

**Supplementary File 5.**

**Table 1.** ActivPAL wear time compliance at baseline and follow-up assessments for the study cohort (n = 162)

| **Wear Time Compliance** | **Baseline (n)** | **Follow-up (n)** | | | **Both baseline and follow-up measures (n)** | |  |
| --- | --- | --- | --- | --- | --- | --- | --- |
| ≥ 1 day | 122 | | 100 | 84 | | 51.85% | |
| ≥ 2 days | 115 | | 92 | 76 | | 46.91% | |
| ≥ 3 days | 106 | | 84 | 67 | | 41.36% | |
| ≥ 4 days | 97 | | 77 | 60 | | 37.04% | |
| ≥ 1 weekday | 122 | | 98 | 83 | | 51.23% | |
| ≥ 1 weekday & 1 weekend | 101 | | 80 | 61 | | 37.65% | |
| ≥ 1 weekday & 2 weekend | 85 | | 72 | 49 | | 30.25% | |
| ≥ 2 weekdays | 111 | | 89 | 73 | | 45.06% | |
| ≥ 2 weekdays & 1 weekend | 97 | | 77 | 67 | | 41.36% | |
| ≥ 2 weekdays & 2 weekend | 84 | | 70 | 48 | | 29.63% | |
| ≥ 3 weekdays | 96 | | 75 | 58 | | 35.80% | |
| ≥ 3 weekdays & 1 weekend | 88 | | 70 | 51 | | 31.48% | |
| ≥ 3 weekdays & 2 weekend | 88 | | 65 | 42 | | 25.93% | |
| ≥ 4 weekdays | 75 | | 62 | 41 | | 25.31% | |
| ≥ 4 weekdays & 1 weekend | 73 | | 60 | 39 | | 24.07% | |
| ≥ 4 weekdays & 2 weekend | 68 | | 55 | 34 | | 20.99% | |
| ≥ 5 weekdays | 5 | | 0 | 0 | | 0.00% | |
| ≥ 5 weekdays & 1 weekend | 5 | | 0 | 0 | | 0.00% | |

Participants were included in the main analysis if they wore the monitor for at least 10 waking hours on at least 2 days (weekday or weekend), at baseline and follow-up (week 8).

**Table 2.** Results (Median, IQR) of Kidscreen-27 between intervention and control groups

|  | **Control** | | **Intervention** | | **INT vs. control*** |
| --- | --- | --- | --- | --- | --- |
| Kidscreen-27 T-scores | **Baseline**  **(n = 68)** | **Follow-up**  **(n = 57)** | **Baseline**  **(n = 68)** | **Follow-up**  **(n = 67)** | β **(95% CI)** |
| Physical wellbeing | 50 (45, 56) | 50 (45, 56) | 52 (44, 59) | 52 (50, 59) | 4.5 (-0.87, 9.90) |
| Psychological wellbeing | 52 (38, 56) | 48 (39, 60) | 47 (38, 56) | 53 (44, 64) | 5.2 (-2.15, 12.51) |
| Autonomy & parents | 47 (39, 63) | 48 (41, 56) | 47 (41, 51) | 48 (43, 59) | 1.0 (-6.57, 8.60) |
| Social support & peers | 53 (47, 66) | 53 (50, 66) | 53 (49, 66) | 58 (50, 66) | 1.4 (-3.50, 6.23) |
| School environment | 56 (45, 58) | 58 (51, 63) | 58 (50, 63) | 58 (53, 71) | 1.1 (-4.21, 6.44) |

Children’s health-related quality of life was assessed via a validated self-report Kidscreem-27 questionnaire (Ravens-Sieberer 2007).

*Between group comparison N = 62; Control = 34; intervention = 28.
