# Supplementary material for: The Children – Sit Less, Move More (C-SLAMM) pilot intervention: Feasibility and acceptability of a multi-component school and home-based intervention to promote physical activity
Source: PLoS One. 2025 Nov 19;20(11):e0335933. doi: 10.1371/journal.pone.0335933 (PMC12629496; doi:10.1371/journal.pone.0335933)
Supplement: S7 File — (PDF) [file pone.0335933.s007.pdf]

Appendix 1 – Study Protocol

Project Title: Children - Sit Less, Move More (C-SLAMM): a pilot study to increase physical activity and decrease sedentary behaviour in primary-school children.

Chief investigator: Prof Marie Murphy (UU)
PhD researcher: Sarah Nally
Other investigators: Prof Alison Gallagher, Dr Angela Carlin, Prof Jo Salmon

Background

Regular physical activity (PA) is associated with a variety of health benefits for children (Warburton *et al.* 2006) and averts the onset of a range of non-communicable diseases, including obesity, type II diabetes and cardiovascular disease (Jeon *et al.* 2007; Lee *et al.* 2012). Despite these well-established health outcomes, data from the United States (Guthold *et al.* 2018), United Kingdom (Townsend *et al.* 2015) and Australia suggest only a third of primary school aged children meet the current international public health PA recommendations of an average of 60 minutes of moderate-to-vigorous physical activity (MVPA) per day (WHO 2020). Furthermore, an all-Ireland study investigating *Children's Sport Participation and Physical Activity*, found that only 17% of primary school children in the Republic of Ireland (ROI) and 20% of primary school children in Northern Ireland (NI) meet the recommended 60 minutes of MVPA a day (CSPPA 2018).

Emerging evidence suggests that, independent of PA, prolonged sitting time adversely affects children's health (Mitchell and Byun 2014). Sedentary behaviour (SB) has been associated with adverse cardio-metabolic heath risk-markers, reduced self-esteem, and academic performance in *children (Tremblay et al.* 2011). SB is the most prevalent health-related behaviour exhibited by children (Abbott *et al.* 2013). To reduce these health risks, recent global recommendations suggest that children should minimise sedentary time and break up sitting time as often as possible (Carson *et al.* 2016). Despite these recommendations, UK children spend more than 65% of waking hours sedentary (Steele *et al.* 2009). Both SB and PA patterns track from childhood into adulthood and reap dividends later in life (Biddle *et al.* 2010; Telama *et al.* 2014). Therefore, the development of effective strategies and early intervention to reduce SB and promote PA is imperative for the current and future health of young people (Farooq *et al.* 2017).

Many PA interventions in children have focused exclusively on individual approaches which aim to encourage children to participate. Despite years of such intervention research, increases in PA remain both modest and short term. There is now widespread recognition that health behaviours are difficult to change, especially in an environment that does not support change, PA and SB interventions have shifted focus to include the factors that influence these behaviours beyond individual choice alone. The socioecological

model recognises that efforts to change health behaviours are more likely to be successful when multiple levels of influence including individual, social, environmental and policy levels are addressed at the same time. To be successful, future intervention development needs to address these multiple influences on behaviour.

Primary schools are an ideal setting to promote PA (Kriemler *et al.* 2011) and reduce SB (Hegarty *et al.* 2016) as children spend 40% of their time at school (Fox 2004). In a recent systematic review, Messing *et al.* (2019) examined the best strategies to promote PA across the settings of family and home, childcare and school. The review found that multi-component interventions stood out prominently and are proven to be the most effective form to promote PA in a school setting. A classroom-based study conducted in children attending primary schools in NI, found that a 5-minute activity break, implemented three times per day for 12 weeks, increased minutes of MVPA accumulated during weekdays (Drummy et al. 2016). Several existing systematic reviews and meta-analysis have analysed the school as a setting and examined the effectiveness of interventions promoting PA within the school-setting (Salmon *et al.* 2007; Dobbins *et al.* 2013; Kriemler *et al.* 2011; Drummy *et al.* 2016; Hegarty *et al.* 2016; Jones *et al.* 2019) and during particular times of the school day, including recess (Ridgers *et al.* 2012), physical education lessons (Lonsdale *et al.* 2013), integration of PA into the curriculum and after-school programs (Mears 2016). Thus, schools provide potentially useful settings to implement strategies designed to increase children's PA and decrease SB.

Transform-Us! is a multi-component school-and family-based intervention which was developed through a substantive series of extensive iterative work in Australia, and which is currently being evaluated in 8-9-year-old Australian children (Salmon *et al.* 2011). Unique to the intervention, and key to its ethos, is its approach to delivery of school curriculum and homework in less sedentary and more active ways, with the overarching aim being to develop an effective and sustainable 'programme' (rather than an 'intervention') in the longer-term. The study aims to increase children's PA and decrease SB across the school day incorporating a mixture of educational, pedagogical, behavioural, and environmental approaches to integrate movement into everyday class lessons, recess/lunchtime, and homework.

Although *Transform-Us*! intervention has proved promising as a multi-component programme and is in the final stages of longer-term evaluation in Australia, we do not have similar multi-component programmes in the UK which have been subjected to rigorous evaluation within the UK or NI. Several local contextual differences between Australia and NI, in particular the educational system, social and environmental influences on PA and policy level differences mean that wholescale replication would be inappropriate. This pilot study will address this gap in the evidence. By using the established framework developed by

Transform-Us! and with direct supervisory input from its creator, Professor Jo Salmon from Deakin University this pilot study will be able to develop and test a bespoke intervention in NI primary schools.

Study aims:

The primary aim of this study is to assess the feasibility/pilot of an 8-week, cluster RCT multicomponent school and family-based intervention to reduce sedentary behaviour and increase physical activity in children aged 7-9 years old . To inform such a trial key information needs to be established around school and participant recruitment, acceptability of the intervention and outcome measures, and attrition rates.

Background to intervention development

This trial has been adapted for the existing *Transform-Us!* intervention which has been piloted and tested in this age group. *Transform-Us!* is currently being evaluated and is available to all primary schools in the state of Victoria, Australia. C-SLAMM is a feasibility study for a clustered RCT of an 8-week school and family-based intervention (with randomisation at school level). The intervention is, adapted from the previously tested *Transform-Us!* trial and informed by a qualitative study was on the perceived barriers and facilitators of physical activity within the classroom, school and/or home environment (REC/20/0033).

Study design

The design conduct and reporting of the intervention with adhere to the Consolidation Standards of Reporting Trials (CONSORT) guidelines and is guided by the Standard Protocol Items for Randomized Trials (SPIRIT) Statement. The intervention will be conducted in line with Medical Research Council guidelines developing and evaluating complex interventions (Craig *et al.* 2008). The RE-AIM framework will also be used in the planning and evaluation stages of the intervention (Glasgow *et al.* 1999).

Intervention

School consenting to participation will be randomly assigned to one of two arms: (1) PA and reduced SB (intervention arm), or (2) current practice (control arm). Pupils (P4 and P5 children) in each school will be recruited to participate in an assessment of physical activity and sedentary behaviour before and after the intervention. Baseline measurements will precede randomisation. Quantitative and qualitative methods will be conducted throughout the intervention period as part of the process evaluation. A process evaluation will focus on factors affecting intervention fidelity, participation, motivation to participate and issues that might affect dissemination.

Piloting some of the elements

Researchers will visit schools on one occasion ahead of commencing the intervention to ensure the intervention components fits within the NI classroom. The aim of this visit will be to avoid encountering any problems within the feasibility pilot study and to ensure the standing desks, easels and active breaks are possible within the classroom setting. The teacher will be asked to provide informal feedback regarding the resources and the practicalities of the intervention components within the classroom. No pupils will be involved at this stage of the research as the focus at this point is solely on the classroom environment.

Setting

This study will be conducted in primary schools in NI. A convenience sample of primary schools in NI will be invited to take part in the study. Eight primary schools (4 intervention, 4 control), each with at least 16 children enrolled in the P4 or P5 year group (approximately 50% of a typical class) will be recruited giving a minimum total sample of 128. According to the Department of Education, class sizes for pupils in P4/P5 should not exceed 30, giving a maximum total sample 240. Consequently, the sample will be sufficiently large to provide clear estimates of recruitment and follow-up for a definitive trial. Children who attend a school selected to take part in the study and are 7-9 years (i.e. in P4/5), who are healthy and free from any medical condition that limits their participation in a physical activity intervention, will be eligible for inclusion in this study.

Recruitment

Principals from eligible schools will be invited to participate initially via telephone or email (Appendix 13). A researcher (SN) will explain the requirements of study participation. Schools will be invited to complete an agreement form to indicate if they are able to participate. Schools participating in another physical activity intervention will be excluded. Once the principal has agreed to take part in the study, teachers will be given an information sheet (Appendix 4) and written consent for participation will be obtained from teachers of P4 and P5 classes (Appendix 8). Then, all children at the participating schools will be provided with an information pack containing a participant information sheet (Appendix 3) and consent form (Appendix 6) to be given to following permission from the school principal. All pupils will be invited to take part in the study. Parents/guardians will be provided with a participant information leaflet (Appendix 5) asked to provide consent (Appendix 7). As the school will have consented to the programme being delivered to all P4 or P5 children, and the programme will be delivered by classroom teachers as part of their daily classroom activities, consent from parents will only be required for the evaluation components of the study. Thus, all children in participating classes will join in the intervention as part of their normal classroom activity; however, data will only be collected from children with parental consent as part of the

evaluation. Additionally, no child will feel excluded, all children in participating classes will be invited to participate.

Randomisation

Following completion of baseline measurements, participants will be randomly allocated by school, using a computer-based random number generator to either receive the intervention or to act as control. Schools will be randomised at the school level (to avoid potential for contamination) to either the intervention or control group post baseline assessments. Each school will be given an anonymous code and randomly allocated to one of two study arms until all 8 schools had been assigned a study arm (i.e. 4 intervention schools, 4 control schools). The procedure will be performed by a researcher at the University, independent of the project. The study arms relating to each randomly allocated number will be written down by the individual before randomisation and concealed in an envelope until all enrolled participants completed all baseline assessments and it was time to allocate the intervention. The individual responsible for randomisation will then notify the research team. Given the nature of the intervention, blinding of schools and participants will not be possible following randomisation. The researcher responsible for subsequent data collection and analysis will not be blinded to group allocation. Any differences between the intervention and control groups will be by chance and adjusted for in the analyses. Schools randomised to control will be offered information on the intervention and associated materials at the end of the intervention.

Intervention

The study will use innovative behavioural, pedological, and environmental strategies within the classroom, school, and home settings to get students moving more and sitting less. Figure 1 shows the study flow diagram. The strategies will be based on strategies used in *Transform-Us!* involve incorporating movement into everyday class lessons – the delivery of the lesson changes, not the content. Resources include health lessons, active lessons, active breaks, active homework to do with parents, active environments, and newsletters for parents. A summary of the intervention components is shown in Table 1. The research team has access to all the *Transform-Us!* materials and the qualitative study has informed the suitability of the materials. Any potential variations in use of language will be revised.

Teachers will be asked to use a logbook to track progress of the intervention. A researcher or a member of staff from the schools will complete an inventory of physical activity and play equipment prior to randomisation. Teachers are responsible for intervention delivery. Teacher training will be provided after randomisation has occurred. Teachers who have been allocated to the intervention group will take part in

the teacher training; the control group will resume normal practice. Teachers will be required to undergo face-to-face teacher training during the normal school day, which will last no more than 2 hours, on a date and time convenient to them. Teachers within the intervention schools will be supplied with online materials after the randomisation has occurred, to ensure no contamination between intervention and control groups. Teacher training will cover what resources are available, how to utilise the resources and how often each intervention component will occur across the school week. Resources include health lessons, active lessons, active breaks, active homework, active environments, and newsletters for parents. Teachers will be provided with four complete lesson (30 minutes in duration) plans and will be encouraged to modify the materials to suit their class and teaching style.

Each week teachers will be advised to incorporate elements of the intervention within the classroom setting. Teachers will modify the delivery of at least one class lesson per week (~30 minutes) so that children will complete the lesson standing up. Teachers will be provided with a suite of standing lesson delivery methods that can be modified to any class topic. If administered as intended, this should result in approximately 150-minutes less sitting time per week. In addition, every two-hour classroom teaching block will be interrupted every 30 minutes with a 2-minute guided light-intensity activity break. This will equate to a total of six minutes interrupted sitting time every two hours. In addition, each class will be provided with six standing desks so that children can rotate learning activities at 'standing stations'. A novelty timer will be given to each class so that teachers can monitor 2-minute standing breaks and every 30-minutes of sitting class time. Two newsletters will be sent home to parents providing project updates and tips on promoting their child's physical activity and how to decrease sedentary behaviour. Physical activity will be promoted and encouraged during recess and lunch breaks. Schools will be provided with sports equipment to make available for children to use in recess and lunch breaks, and teachers and peers will provide encouragement and support for active games. Homework tasks will be modified to incorporate physical activity and children will be encouraged to complete these tasks with their parents (e.g., go for a walk with their parents and write about where they went and what they saw; mathematics homework using their stride as the unit of measurement). Examples of some of the resources that will be used in the C-SLAMM intervention are included in Appendix 9. All the materials and resources used have been tried and tested in this age group.

Control arm

To compare the effects of the intervention against usual practice, schools assigned to the control arm will be requested to continue with their usual practice and lesson delivery, no environmental changes will be made to their classrooms. The participants in the control schools will be asked to complete the same study measurements as those in the intervention schools at the same time points. Upon completion of the study,

control schools will receive a report summarising their pupils' sitting and physical activity data. They will also receive adapted materials upon completion of all follow-up evaluation measures.

Table 1 Components of the intervention

**Intervention component**	**Setting**	**Element**	**Description**
Curriculum component	Classroom	Health lessons with key learning messages	1. Key messages will focus on raising awareness; self-monitoring; goal setting; behavioural contracts; social support (team-based activities at school; homework to do with parents); and feedback and reinforcement (external and intrinsic rewards).<br>2. Encouraged to meet the 60 minutes of MVPA per day recommendation and to minimise screen time.
Class strategies	Classroom	Interrupting classroom sitting time	1. Teachers will modify the delivery of one class lesson per day (30 minutes) so that children will complete the lesson standing up.<br>2. Every two-hour classroom teaching block will be interrupted every 30 minutes with a 2-minute guided light-intensity active break.
Physical environment	Classroom	Environmental cues and prompts	Each class will be provided with six adjustable sit-stand desks so that children **can rotate** learning activities at "standing stations".
Physical environment	School	Environmental cues and prompts	1. Provision of sporting equipment, line markings and signage to promote PA<br>2. Provision of pedometers for each class
Homework assignments	Family	Modification of homework	1. Homework tasks will be modified to reduce sitting time and increase activity while completing them at home<br>2. Children will also be given homework tasks to complete with parents.
Newsletter	Family	Dissemination of information	1. The newsletter will support the key learning messages delivered to the children in the classroom.<br>2. Newsletters will incorporate family-based activities for parents to complete with their child and contain information about ways to reduce their child's screen time and increase activity.

Outcomes

Data collection will take place twice. All participating children will be tested at baseline and post intervention (week 8).

Primary outcome measures:

Sedentary time and physical activity

The primary outcome measure will be minutes of light PA, MVPA and minutes of daily sedentary behaviour per weekday measured using an accelerometer to objectively measure physical activity and sedentary behaviour. Participants will be provided with a brief diary during each monitoring period where they will be requested to document time in bed and any periods of non-wear (Appendix 16), thus, sleep time and non-wear time noted in the diary will be removed from analysis. Children will wear the accelerometer continuously (24 h/day) for seven consecutive days during each measurement period. All health-related outcome measurements will be taken twice, at baseline (prior to randomisation) and post-intervention (week 8). The proportion of pupils meeting the recommended amount of PA will be determined at baseline and post-intervention.

Secondary outcome measures:

Anthropometry

Height (cm) and weight (kg) will be measured to the nearest 0.1 cm and 0.1 kg, it will be converted to an age-specific and gender-specific z-score. The child will be asked to face forward, with shoes removed. Anthropometry measures at baseline and at the end of the intervention will include height and weight (BMI). Height (cm) will be measured to the nearest 0.1 cm using a free-standing stadiometer (Leicester Height Measure, Marsden Group, Unit 7, Centurion Business Park, Coggin Mill Way, Rotherham, S60 1FB) and weight (kg) will be measured to the nearest 0.1 kg using digital scales (Seca 877, Medical). These measurements will all be conducted by a trained researcher (SN) in anthropometric techniques. Participants will be provided with the opportunity to remove shoes and jumpers/jackets for these measurements. All measurements will be conducted in a semi-screened area with at least 1 researcher present, with another adult present or in earshot during data collection for example a member of the research team and a member of school staff.

If the intervention is deemed to have a positive impact on PA and SB that a fully powered cluster RCT will be undertaken across the full school year and although we do not expect changes in height, weight or BMI z-scores in this pilot these measures are being included to ensure fidelity/completeness.

Questionnaires: children, parents/guardians

Children's health-related quality of life (HRQoL) will be assessed by self-report using the Kidscreem-27 questionnaire (Ravens-Sieberer *et al.* 2007), which consists of 27 items covering the following five QoL dimensions: physical wellbeing, psychological wellbeing, parents/guardians relations and autonomy, social support and peers, and the school environment (Appendix 14). The Kidscreen-27 questionnaire was developed simultaneously in several European countries and has been validated in UK children aged 8-18 (Ravens-Sieberer *et al.* 2010) and will take children an estimated 10-15 minutes to complete.

Parents will be asked to complete two short questionnaires: a 20-minute parent-focused questionnaire (Appendix 12), which will ask questions regarding physical activity at home, their son/daughters' physical activity, and travel modes and a Kidscreen-27 questionnaire. The Kidscreen-27 questionnaire (Ravens-Sieberer *et al.* 2007), requires only 10-15 minutes to complete and consists of 5 items covering the following five QoL dimensions: physical wellbeing, psychological wellbeing, parents/guardians' relations and autonomy, social support and peers, and the school environment (Appendix 15).

Economic data

Data will be collected to estimate intervention costs to do a rough estimation of cost-effectiveness analysis of a definitive trial (which would then include a full cost effectiveness analyses). Resource use data, including intervention materials, venue costs, staff, trainer and pupil time, expenses, travel, and administration costs, will be collected prospectively during each stage of the intervention.

Process evaluation

All schools will be informed of the process evaluation requirements of being in the intervention group prior to consent. The write draw show and tell (WDST) methodology (Noonan *et al.* 2016) will be used with participating children to elicit their perceptions and experiences of the intervention components. A sub-sample of children (n =6) from each intervention school will be included for the process evaluation. No inclusion/exclusion criteria will be set for children to be included, other than having parental consent to participate. Semi-structured WDST guides will be developed and used to ensure consistency across WDST groups (Appendix 10). Arrangements will be made with relevant staff within each school for the procedures outlined in the section below to be completed during and after the implementation phase of the intervention.

Four one-to-one interviews (one per teacher) (Appendix 11) will be undertaken with the participating teachers during the process evaluation at the end of the intervention. The first author (SN) will conduct these interviews which will be recorded and transcribed. The interviews with teachers will be designed to

identify barriers and facilitators to implementation of the intervention, as well as strengths and weaknesses of the programme.

References for research protocol

Abbott, R.A., Straker, L.M. and Erik Mathiassen, S. (2013), Patterning of children's sedentary time at and away from school. *Obesity*, 21: E131-E133. https://doi.org/10.1002/oby.20127

Bull, F.C., Al-Ansari, S.S., Biddle, S., Borodulin, K., Buman, M.P., Cardon, G. *et al...* Chou, R. (2020) World Health Organization 2020 guidelines on physical activity and sedentary behaviour. *British Journal of Sports Medicine,* 54(24), 1451-1462.

Carson, V., Hunter, S., Kuzik, N., Gray, C.E., Poitras, V.J., Chaput, J. *et al...* Tremblay, M.S. (2016) Systematic review of sedentary behaviour and health indicators in school-aged children and youth: an update. *Applied Physiology, Nutrition, and Metabolism,* 41(6), S240-S265. https://doi.org/10.1139/apnm-2015-0630

Craig P, Dieppe P, Macintyre S, Michie S, Nazareth I, Petticrew M; Medical Research Council Guidance. (2008) Developing and evaluating complex interventions: the new Medical Research Council guidance. *British Medical Journal*. 337:a1655. https://doi.org/10.1136/bmj.a1655

Dobbins M, Husson H, DeCorby K, et al. (2013) School-based physical activity programs for promoting physical activity and fitness in children and adolescents aged 6 to 18. *Cochrane Database Systematic Reviews*, 2, CD007651.

Ding, D., Mutrie, N., Bauman, A., Pratt, M., Hallal, P.R.C. and Powell, K.E. (2020) Physical activity guidelines 2020: comprehensive and inclusive recommendations to activate populations. *The Lancet*, 396(10265), 1780-1782. https://doi.org/10.1016/S0140-6736(20)32229-7

Farooq, M., Parkinson, K., Adamson, A., Pearce, M., Reilly, J., Hughes, A., Janssen, X., Basterfield, L. and Reilly, J. (2017). Timing of the decline in physical activity in childhood and adolescence: Gateshead Millennium Cohort Study. *British Journal of Sports Medicine*, 52(15), pp.1002-1006.

Fox, K., Cooper, A. and McKenna, J. (2004). The School and Promotion of Children’s Health-Enhancing Physical Activity: Perspectives from the United Kingdom. *Journal of Teaching in Physical Education*, 23(4), pp.338-358.

Glasgow RE, Vogt TM, Boles SM. (1999). Evaluating the public health impact of health promotion interventions: the RE-AIM framework. *American Journal of Public Health*, 89(9), 1322-1327.

Guthold, R., Stevens, G.A., Riley, L.M. and Bull, F.C. (2018) Worldwide trends in insufficient physical activity from 2001 to 2016: a pooled analysis of 358 population-based surveys with 1·9 million participants. *The Lancet Global Health,* 6(10), e1077-e1086. https://doi.org/10.1016/S2214-109X(18)30357-7

Jeon, C.Y., Lokken, R.P., Hu, F.B. and van Dam, R.M. (2007) Physical activity of moderate intensity and risk of type 2 diabetes: a systematic review. *Diabetes Care,* 30(3), 744-752.

Julious, S.A. (2005) Sample size of 12 per group rule of thumb for a pilot study. *Pharmaceutical Statistics,* 4(4),287-291.

Jones, M., Defever, E., Letsinger, A., Steele, J. and Mackintosh, K. (2019). A mixed-studies systematic review and meta-analysis of school-based interventions to promote physical activity and/or reduce sedentary time in children. *Journal of Sport and Health Science.*

Kriemler, S., Meyer, U., Martin, E., van Sluijs, E., Andersen, L. and Martin, B. (2011). Effect of school-based interventions on physical activity and fitness in children and adolescents: a review of reviews and systematic update. *British Journal of Sports Medicine*, 45(11), pp.923-930.

Lonsdale, C., Rosenkranz, R., Peralta, L., Bennie, A., Fahey, P. and Lubans, D. (2013). A systematic review and meta-analysis of interventions designed to increase moderate-to-vigorous physical activity in school physical education lessons. *Preventive Medicine*, 56(2), pp.152-161.

Lee, I., Shiroma, E.J., Lobelo, F., Puska, P., Blair, S.N. and Katzmarzyk, P.T. (2012) Effect of physical inactivity on major non-communicable diseases worldwide: an analysis of burden of disease and life expectancy. *The Lancet,* 380(9838),219-229. https://doi.org/10.1016/S0140-6736(12)61031-9

Mears, R. and Jago, R. (2016). Effectiveness of after-school interventions at increasing moderate-to-vigorous physical activity levels in 5- to 18-year olds: a systematic review and meta-analysis. *British Journal of Sports Medicine*, 50(21), pp.1315-1324.

M. Hegarty, L., L. Mair, J., Kirby, K., Murtagh, E. and H. Murphy, M. (2016). School-based Interventions to Reduce Sedentary Behaviour in Children: A Systematic Review. *AIMS Public Health*, 3(3), pp.520-541.

Mitchell, J.A. and Byun, W. (2014) Sedentary Behavior and Health Outcomes in Children and Adolescents. *American Journal of Lifestyle Medicine,* 8(3), 173-199. https://doi.org/10.1177/1559827613498700

Morgan PJ, Young MD, Smith JJ, Lubans DR (2016). Targeted health behavior interventions promoting physical activity: A conceptual model. *Exercise and Sport Sciences Reviews*, 44(2), 71-80.

Murray, D.M. (1998) *Design and analysis of group-randomized trials.* Oxford University Press, USA.

Noonan, R., Boddy, L., Fairclough, S. and Knowles, Z. (2016). Write, draw, show, and tell: a child-centred dual methodology to explore perceptions of out-of-school physical activity. BMC Public Health, 16(1). Punch, S. (2002). Research with children: The same or different from research with adults? *Childhood*, 9(3), pp.321-341.

Ravens-Sieberer, U., Auquier, P., Erhart, M. et al. The KIDSCREEN-27 quality of life measure for children and adolescents: psychometric results from a cross-cultural survey in 13 European countries. *Qual Life Res* 16, 1347–1356 (2007). https://doi.org/10.1007/s11136-007-9240-2

Reiner, M., Niermann, C., Jekauc, D. and Woll, A. (2013). Long-term health benefits of physical activity – a systematic review of longitudinal studies. *BMC Public Health*, 13(1).

Ridgers, N., Salmon, J., Parrish, A., Stanley, R. and Okely, A. (2012). Physical Activity During School Recess. *American Journal of Preventive Medicine,* 43(3), pp.320-328.

Ridgers ND, Timperio A, Cerin E, Salmon J. (2014) Compensation of physical activity and sedentary time in primary school children. *Medicine and Science in Sport and Exercise*. 46(8):1564-9.

Rowland TW. (1998) The biological basis of physical activity. *Medicine and Science in Sport and Exercise*. 30(3):392–9.

Salmon, J., Booth, M., Phongsavan, P., Murphy, N. and Timperio, A. (2007). Promoting Physical Activity Participation among Children and Adolescents. *Epidemiologic Reviews*, 29(1), pp.144-159.

Salmon J, Arundell L, Hume C, Brown H, Hesketh K, Dunstan DW, Daly RM, Pearson N, Cerin E, Moodie M, Sheppard L, Ball K, Bagley S, Paw MC, Crawford D. (2011) A cluster-randomized controlled trial to reduce sedentary behavior and promote physical activity and health of 8-9 year olds: the Transform-Us! study. *BMC Public Health*. 11:759.

Telama, R., Yang, X., Leskinen, E., Kankaanpaa, A., Hirvensalo Irvensalo, M., Tammelin, T., Viikari, J. and Raitakari, O. (2014). Tracking of Physical Activity from Early Childhood through Youth into Adulthood. *Medicine & Science in Sports & Exercise*, 46(5), pp.955-962.

Tremblay, M., LeBlanc, A., Kho, M., Saunders, T., Larouche, R., Colley, R., Goldfield, G. and Gorber, S. (2011). Systematic review of sedentary behaviour and health indicators in school-aged children and youth. *International Journal of Behavioral Nutrition and Physical Activity*, 8(1), p.98.

Verloigne, M., Loyen, A., Van Hecke, L., Lakerveld, J., Hendriksen, I., De Bourdheaudhuij, I., Deforche, B., Donnelly, A., Ekelund, U., Brug, J. and van der Ploeg, H. (2016). Variation in population levels of sedentary time in European children and adolescents according to cross-European studies: a systematic literature review within DEDIPAC. *International Journal of Behavioral Nutrition and Physical Activity*, 13(1).

Warburton, D.E.R., Nicol, C.W. and Bredin, S.S.D. (2006) Health benefits of physical activity: the evidence. *Cmaj*, 174(6), 801. https://doi.org/10.1503/cmaj.051351

Woods, C, Powell, C, Saunders, JA, O'Brien, W, Murphy, MH, Duff, C, Farmer, O, Johnston, A, Connolly, S, Belton, S & Belton, S 2019, The Children’s Sport Participation and Physical Activity Study 2018 (CSPPA 2018): Final Report. Sport Ireland, Dublin, Ireland.
